# The ResidentialCare Transition Module: a single-blinded randomized controlled evaluation of a telehealth support intervention for family caregivers of persons with dementia living in residential long-term care

**DOI:** 10.1186/s12877-020-01542-7

**Published:** 2020-04-15

**Authors:** Joseph E. Gaugler, Tamara L. Statz, Robyn W. Birkeland, Katie W. Louwagie, Colleen M. Peterson, Rachel Zmora, Ann Emery, Hayley R. McCarron, Kenneth Hepburn, Carol J. Whitlatch, Mary S. Mittelman, David L. Roth

**Affiliations:** 1 Division of Health Policy and Management, School 8of Public Health, University of Minnesota, D351 Mayo (MMC 729), 420 Delaware Street S.E, Minneapolis, MN 55455 USA; 2 Division of Epidemiology and Community Health, School of Public Health, University of Minnesota, Minneapolis, MN USA; 3 Nell Hodgson Woodruff School of Nursing, Emory University Alzheimer’s Disease Research Center, Emory University, Atlanta, GA USA; 4 Benjamin Rose Institute on Aging, Cleveland, OH USA; 5 Department of Psychiatry, NYU School of Medicine, NYU Langone Health, New York, NY USA; 6 Center on Aging and Health, Johns Hopkins University, Baltimore, MD USA

**Keywords:** Institutionalization, Nursing home admission, Nursing home placement, Nursing home entry, Caregiving, Informal care, Intervention, Familywor

## Abstract

**Background:**

Families do not fully disengage from care responsibilities following relatives’ admissions to residential long-term (RLTC) care settings such as nursing homes. Caregiver stress, depression, or other key outcomes remain stable or sometimes increase following a relative’s RLTC entry. Some interventions have attempted to increase family involvement after institutionalization, but few rigorous studies have demonstrated whether these interventions are effective in helping families navigate the potential emotional and psychological upheaval presented by relatives’ transitions to RLTC environments. The Residential Care Transition Module (RCTM) provides six formal sessions of consultation (one-to-one and family sessions) over a 4-month period to family caregivers who have admitted a relative to a RLTC setting.

**Methods:**

In this embedded mixed methods randomized controlled evaluation, family members who have admitted a cognitively impaired relative to a RLTC setting are randomly assigned to the RCTM (*n* = 120) or a usual care control condition (*n* = 120). Primary outcomes include reductions in family members’ primary subjective stress and negative mental health outcomes; secondary role strains; and residential care stress. The mixed methods design will allow for an analysis of intervention action mechanisms by “embedding” qualitative components (up to 30 semi-structured interviews) at the conclusion of the 12-month evaluation.

**Discussion:**

This evaluation will fill an important clinical and research gap by evaluating a psychosocial intervention designed for families following RLTC admission that determines whether and how the RCTM can help families better navigate the emotional and psychological challenges of residential care transitions.

**Trial registration:**

ClinicalTrials.gov (NCT02915939, prospectively registered).

## Background

The Residential Care Transition Module (RCTM) is a psychosocial (provision of counseling and support) and psychoeducational (delivery of skills building and strategies) intervention designed to help families successfully adapt to the admission of a cognitively impaired relative to a residential long-term care (RLTC) facility. Residential long-term care facilities in the RCTM include nursing homes (NHs), memory care units in assisted living settings, or similar environments. The RCTM transition counselors (TCs) provide six individualized formal sessions to family members over a 4-month period. Together, TCs and family caregivers identify individual placement stressors and enhance caregivers’ strategies for coping with them.

Persons with dementia rely heavily on informal (i.e., unpaid) sources of care. Currently, 83% of the 5.8 million persons with Alzheimer’s disease or a related dementia (ADRD) in the U.S. are cared for by one or more family members and over 16 million individuals provide unpaid care to persons with ADRD in the U.S [1]. A well-established literature demonstrates the adverse effects of dementia caregiving on family members, including impaired physical health, financial strain, degradation in social well-being, and increased prevalence of depression, anxiety, or other psychological symptoms [2–5].

Longitudinal analyses of dementia caregiving make it clear that caregiving does not end with the institutionalization of a cognitively impaired relative [6]. The high prevalence of dementia among NH residents (50% of NH residents have ADRD and 61% have moderate or severe cognitive impairment) [7, 8] likely influences the need for at least some ongoing family care. Family members thus remain engaged in the lives of institutionalized relatives [6, 9]. Direct care workers in NHs or other residential care settings often assume responsibility for personal care. However, families remain involved in other aspects of help following relatives’ RLTC entry, including emotional support and social engagement, instrumental assistance (e.g., help with financial management), and advocating for quality care from the RLTC setting and staff [6, 9–12]. Several studies suggest that dementia caregiver distress remains stable or may even increase following care recipients’ admission to RLTC [13–17]. Caregivers with their own health challenges in particular appear susceptible to high burden and depression up to 12 months following cognitively impaired relatives’ institutionalization [18, 19]. Psychosocial and psychoeducational interventions could ease the RLTC transition for caregivers and alleviate these potentially adverse outcomes.

Facilitating family caregivers’ RLTC transitions is important because their well-being may influence their relative’s quality of life once in a residential setting. Various studies have emphasized that while NHs are oriented to delivering the necessary physical care, these facilities often fall short of providing hospitable environments or encouraging individual residents to pursue the goal of a “life worth living” [20–24]. Several studies imply that social engagement, family visits, and other types of involvement can potentially improve life satisfaction and health outcomes for NH or assisted living residents [25–29]. These findings suggest that reducing emotional distress and negative mental health outcomes and enhancing families’ overall perceptions of and relationships with staff can have positive effects on residents’ outcomes.

There is a demonstrable need for supporting family members following placement of a relative in RLTC. Scholars emphasize the importance of incorporating families in the provision of services and care to cognitively impaired older adults in residential care settings [30]. However, most services for families are designed for at-home caregivers [31], and in prior intervention studies RLTC placement has been conceptualized as an outcome to be prevented or delayed. Earlier RLTC-based interventions that sought to increase the frequency and quality of family involvement (e.g., reduce staff-family conflict) can be categorized into three models: group protocols that include peer-led support [32–36], limited telephone-based counseling support to families [37], and staff-family partnerships that attempt to clarify family and staff roles and responsibilities in RLTC [38–41]. These various approaches have modest, mixed, or no effects in increasing family involvement, enhancing staff satisfaction, and improving resident well-being [42]. Although several pilot studies and a published randomized controlled trial (RCT) report on providing direct support to families of institutionalized relatives, these programs either lack sufficient rigor to support their implementation or did not result in positive outcomes for family caregivers due in part to the clinical content, delivery, and measurement approach selected [43–45]. The RCTM will fill an important clinical and research gap, as it is a multidimensional intervention designed specifically for families following RLTC entry of a relative. This protocol paper outlines the procedures we will employ to determine whether and how the RCTM can help families better navigate residential care transitions of cognitively impaired relatives.

The conceptual model underlying the evaluation of the RCTM is the Stress Process Model for Residential Care (SPM-RC), developed by Whitlatch and colleagues [16] and based on the widely used Stress Process Model (SPM) for dementia caregiving [46]. The SPM provides a conceptual framework explaining how dementia caregiving stress influences key outcomes throughout the dementia care trajectory [46, 47]. Specifically, the SPM postulates that the frequency and duration of care demands (e.g., activity of daily living dependencies; behavioral challenges; cognitive impairment) adversely influence caregivers’ appraisals of these demands via elevated subjective stress (e.g., emotional exhaustion; feelings of being trapped in the caregiving role). As subjective stress exacerbates, caregivers’ other life domains (family relationships; work/life balance) and their global well-being are threatened (e.g., caregiver self-rated health; depressive symptoms). Stress appraisals’ negative influence on life domains beyond dementia care and overall health is called proliferation [47]. The socidemographic context of care may moderate proliferation. In addition, psychosocial and instrumental support offered through formal or informal channels may limit proliferation by helping dementia caregivers re-appraise care demands, develop strategies to prevent or manage care demands, or offer respite from care responsibilities, thus improving caregivers’ overall well-being.

The SPM-RC adds and refines several interconnected domains of the SPM to result in a model that is directly pertinent to RLTC. One domain centers on emotional and interpersonal family relationships; another focuses on families’ relationships with RLTC staff; another encompasses families’ relationships with the RLTC; and a fourth examines the care setting itself. The SPM-RC captures the possible changes in relationship processes and structures in areas such as the emotional closeness between the relative and family member and family members’ perceptions of difficulty when managing relatives’ emotional and mental status (an appraisal that may change and expand once a relative enters a RLTC setting). Family members’ perceptions of their relative’s adjustment to the RLTC setting can produce “secondary” role strain, particularly if the family member feels guilt or believes the placement decision is contrary to the wishes of the relative. With RLTC admission, an array of placement-related stressors may emerge. These stressors include challenges that caregivers may experience when establishing effective roles and relationships with direct care workers or other facility staff, attempting to remain involved in the life of the relative in order to maintain or improve quality of life, and advocating for more appropriate care if a deficit in the quality of institutional care is perceived [48, 49]. Personal and organizational stressors frequently interact: if family members perceive that their relative is not doing well, they may increase their engagement through advocacy, hands-on care, or other involvement in order to improve their relative’s overall sense of well-being [29, 40, 48, 49]. Similarly, family members’ own perceptions of how they have adjusted to a relative’s placement and the potentially new roles they have assumed may contribute to their stress. The SPM-RC includes contextual indices related to family caregivers’ interactions with and perceptions of the RLTC setting itself. Family members’ appraisals of their involvement with and the quality of interactions with staff may reflect how well family members’ perceive their own – as well as their relative’s – overall adjustment to the residential care setting. The SPM-RC model incorporates these additional stressors to capture the experience of RLTC for family members, including the influence of this transition on key emotional and mental health outcomes. Prior efforts have successfully analyzed and tested the SPM-RC model that informs the RCTM intervention and the several hypothesized relationships within it [16].

## Methods

This protocol adhered to SPIRIT guidelines/methodology [50]. The SPIRIT flow diagram of the RCTM is presented in Table 1. As of February 2020, this study has completed recruitment (*n =* 240 of planned enrollment of 240). We anticipate completing follow-up data collection in March, 2021. The University of Minnesota Institutional Review Board (UMN IRB; #1511S80406) has approved this protocol. In addition to ongoing review and approval of protocol modifications, the UMN IRB provides continuing approval to the protocol annually. These annual reports include information about participant accrual and withdrawal. Data safety monitoring reports are submitted annually to the project sponsor, the National Institute on Aging (see below).

**Table 1:** SPIRIT Participant Timeline Diagram

	STUDY PERIOD					
	*Enrollment*	*Allocation*	*Post-Allocation*			*Close-Out*
*Time Point*		*Baseline*	*4 Months*	*8 Months*	*12 Months*	*Months 13–15*
ENROLLMENT						
Eligibility Screen	●					
Informed Consent	●					
Allocation		●				
INTERVENTION		●				
Residential Care Transition Module			●	●	●	
Usual care control			●	●	●	
ASSESSMENTS						
*Outcomes*						
Primary: Primary subjective stress during RLTC • Care-related strain • Zarit Burden		●	●	●	●	
Secondary: Secondary role stressors • Relative’s level of adjustment • Caregiver adjustment		●	●	●	●	
Secondary: Residential care stress • Caregiver perception of staff communication with families • Staff support for families • Positive and negative interactions • Family Involvement Interview • Visiting patterns • Frequency of visits		●	●	●	●	
Secondary: Caregiver depressive symptoms • Center for Epidemiological Studies-Depression Scale • Mood Assessment Scale		●	●	●	●	
*Other Variables*						
Context of care • Geographical location • Caregiver and care recipient demographics • Duration and extent of care recipient memory problems • Dementia diagnosis • Medicaid status • Time since RLTC admission		●				
Context of care • Care recipient living arrangement • Rating of RLTC care		●	●	●	●	
Primary objective stressors • Care recipient activity of daily living dependencies • Care recipient instrumental activity of daily living dependencies • Care recipient memory impairment • Revised Memory and Behavior Problems Checklist		●	●	●	●	
Resources • Socioemotional support • Self-rated caregiver health (4 items) • Number and frequency of community-based and health services used • Caregiver activity of daily living dependencies • Caregiver instrumental activity of daily living dependencies • Self-Administered Co-Morbidity Questionnaire • Caregiver self-efficacy • Short Sense of Competence Questionnaire		●	●	●	●	
Semi-structured interviews						●

NOTE: *RLTC* residential long-term care; see text for description of measures and citations

### Aims

This study has the following Aims:

- *Specific Aim 1.* Assess whether the RCTM yields statistically significant (*p* < .05) reductions in caregivers’ primary subjective stress (e.g., burden) and negative mental health outcomes (depressive symptoms) in the 12 months following enrollment when compared to controls;
- *Secondary Aim 1a.* Determine whether the RCTM results in greater increases in caregiver competence and self-efficacy when compared to controls;
- *Secondary Aim 1b.* Ascertain whether those who receive the RCTM report greater family involvement and visits to relatives in RLTC when compared to usual care controls;
- *Specific Aim 2.* Determine whether caregivers who receive the RCTM indicate statistically significant decreases in secondary role strains (perceived adjustment of the relative and the caregiver to RLTC placement) over a 12-month period when compared to caregivers in the usual care control group;
- *Specific Aim 3.* Determine whether caregivers receiving the RCTM report statistically significant decreases in residential care stress (e.g., improved perceptions of staff communication or staff support; reduced upset at having a relative in residential care; reduced negative interactions with relatives or staff in the facility) when compared to caregivers in the usual care control group; and
- *Specific Aim 4.* Delineate the mechanism of action of RCTM under conditions of high and low success by embedding qualitative components (up to 30 semi-structured interviews) at the conclusion of the 12-month evaluation.

### Design

**Fig. 1:**
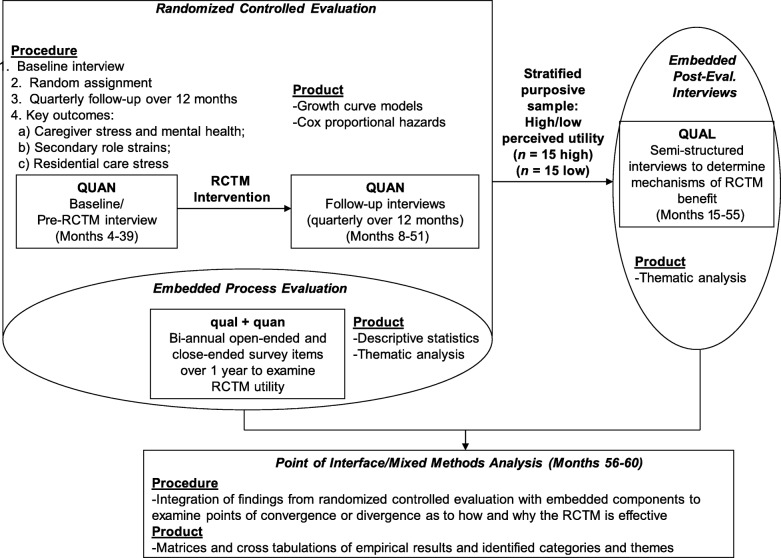
Design of the Residential Care Transition Module evaluation

We employ an embedded experimental mixed methods design to evaluate the RCTM [51]. *Mixed methods* is generally defined as the collection and analysis of both quantitative and qualitative data that link these two forms of data concurrently or sequentially [52]. Data integration can occur within the design of a single study or across multiple studies [51]. Through combination, integration, or comparison of various qualitative and quantitative study “strands,” mixed methods research is typically used to provide greater explanation or expansion of study findings than if only qualitative or quantitative data were collected [53]. The proposed design combines the collection and analysis of qualitative data within a traditional randomized controlled trial design; the collection of embedded qualitative data in this study occurs during and following the randomized controlled trial (see Fig. 1) [51]. The analysis of qualitative data will enhance interpretation of quantitative outcomes and will allow for a more in-depth exploration of the mechanisms and pathways that lead to benefit [51, 54–57].

### Setting

The RCTM is delivered by two trained TCs over the telephone or via secure web-based video conferencing to family caregivers of relatives with ADRD living in a RLTC setting. Family caregivers of persons with ADRD located anywhere in the U.S. can participate. Counseling calls are scheduled based on the family caregiver’s convenience and take place in a secure and private environment at UMN or via secure mobile phones used only by the TCs. Co-Investigators from Johns Hopkins University, New York University Langone Medical Center, Emory University, and the Benjamin Rose Institute on Aging have all collaborated with the UMN team when conceptualizing the RCTM protocol, and will continue to assist on all dissemination efforts. Although Johns Hopkins (the location of the project biostatistician) will collaborate on de-identified data analysis, personnel at the UMN site have oversight of all study procedures, data collection, data management, and dissemination.

### Characteristics of participants

**Fig. 2:**
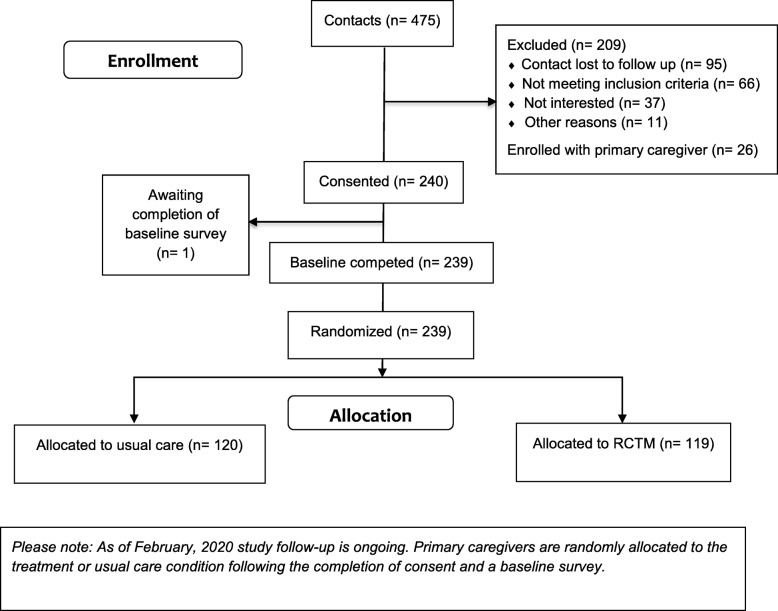
CONSORT Study Diagram

Eligibility criteria include family caregivers who consider themselves the most involved in visiting and providing assistance to the person with memory loss (or who share this primary caregiving role equally). Care recipients must live in a RLTC setting (e.g., assisted living, nursing home, memory care, or other RLTC setting) and have received a physician’s diagnosis of ADRD. Family caregivers must be English speaking, 21 years of age or older, and not participating in any other type of service that provides one-to-one consultation specific to caregiving (participation in general counseling and/or a support group does not prohibit enrollment). Family caregivers using psychotropic medications, such as anti-depressants or anti-psychotics, are eligible if they have remained on a stable dosage for 3 months or longer. The goal of this study is to successfully enroll 240 family caregivers who meet the eligibility criteria; as of February 2020, all 240 caregivers are enrolled. Figure 2 presents a diagram of the study flow.

### Processes, interventions, and procedures

We have adopted a multi-faceted recruitment strategy to identify and enroll eligible participants. The initial recruitment strategy included reaching out to members of LeadingAge (a national advocacy organization for long-term care providers with chapters in each U.S. state) to describe the study, share materials, and encourage staff to distribute recruitment materials (flyers, information sheets, documentation of permission to contact forms) to potentially interested family caregivers. The Principal Investigator (PI) contacted interested LeadingAge members approximately every 6 months to remind them to share study information with staff in their networks. Within Minnesota and in those states where LeadingAge contacts have been particularly responsive, a second recruitment strategy has included placing advertisements in local newspapers/circulars (*N* = 44). In addition, the PI has created a UMN Caregiver Registry that includes family and professional caregivers who have participated in various community education and engagement events he and his research team have offered since 2010. Enrollees in the Registry provide the PI and his research staff permission to contact and invite them to participate in his studies. As of October 2019 the Registry includes 485 family caregivers and 240 professional care providers. In addition to the above methods of recruitment, the research team developed online announcements to allow interested parties to contact the research team directly. These included an online advertisement on the UMN Clinical Translational and Scientific Institute’s StudyFinder page, a project listing on the UMN School of Public Health website, and the use of targeted advertisements on Facebook.

A research coordinator administers a telephone-based screening to determine the eligibility of family caregivers. Following screening, eligible caregivers are invited to complete the consent procedures. Participants complete consent forms electronically or via mailed paper forms. A RC administers all online consent forms via the secure UMN Qualtrics survey application. Following the completion of consent, a RC administers baseline surveys electronically or hard copy via mail. A RC uses Qualtrics to distribute and manage online surveys.

Upon completion of the baseline survey, participants are randomly assigned in a 1:1 ratio to receive either the RCTM intervention or to a control condition by a RC. The project biostatistician generates a random assignment schedule using a random number generator provided by SAS. Treatment condition assignments generated by this program are printed and individual caregiver assignment slips are placed in sequentially numbered, sealed opaque envelopes. The next sealed envelope in the sequence is opened at the time a RC randomly assigns each individual participant. The randomization schedule is stratified by family member relationship (spouse vs. non-spouse) and time since the relative’s admission (3 months or less vs. over 3 months) to ensure balance on these important predictors of caregiver outcomes following RLTC entry. Cases are randomized within variable-sized blocks that range from six to 10 participants. No research staff or investigator interacting with potential participants have access to the random assignment schedule, and because of the variable block sizes and the sealed assignment envelopes, no person with participant contact is able to determine the next treatment assignment in the sequence. These procedures accomplish both random treatment assignment and treatment allocation concealment throughout the trial and are consistent with the recommendations of the CONSORT statement [58]. Following completion of the baseline survey, the RC informs the family caregiver of their randomization status.

To partially adjust for the social engagement provided to the RCTM treatment condition, the TCs complete contact calls with each participant following completion of four, eight, and 12-month surveys. In order to balance ethics with the integrity of the randomized control design, the TCs can provide information and referral (e.g., local phone numbers of the Alzheimer’s Association or an Area Agency on Aging) during these contact calls upon participant request. The TCs can also provide information and referral support if caregivers in the control group initiate contact with the TC for care needs. The TCs collect data on the duration, frequency, and content of each control participant’s contact call.

Table 2 describes the RCTM components’ mode of delivery, frequency, and other essential features [59]. The RCTM incorporates psychosocial and psychoeducational approaches with the objective of: a) focusing on the identification of potential stressors associated with RLTC placement for caregivers; and b) assisting caregivers to develop more effective individual coping strategies and enhanced caregiving self-efficacy following the institutionalization transition. As critical reviews of caregiver interventions suggest, multi-component programs that provide some combination of therapeutic/social support along with training and skills-based modules appear most likely to improve caregiving outcomes [60, 61]. The RCTM similarly offers individual and family counseling, ad hoc support, and knowledge and skills transfer to help families adapt to RLTC [62].

**Table 2:** Residential Care Transition Module Intervention Characteristics

Dimension	Definition	Options
*Delivery Characteristics*		
Mode	Method of contact betweeninterventionist andparticipant	• Telephone contact with primary caregiver only or including other family members at primary caregiver’s discretion • Secure web conferencing with primary caregiver only or including other family members at primary caregiver’s • Email • Text, if preferred by primary caregiver • Mail, if preferred by primary caregiver
Materials	Materials used in thedelivery of the intervention	• Treatment manual • Internet resources, including links to websites, videos, and articles • Printed resource materials for those without internet access
Location	Where the intervention is delivered	• Tele-health: Telephone or secure web conference calls are placed to location of primary caregiver’s preference (often their home; cell phone use is common, so location is frequently varied)
Schedule	Duration and intensity ofintervention	• Six intervention sessions within 1st 4 months • Minutes of contact per session (Duration range: 45–150 min, with an average of 75 min; data current as of 11/18/19) • Dates of intervention sessions (first three occur weekly, next three occur monthly) • Number of ad-hoc sessions (varies; range: 0–38 sessions; data current as of 11/18/19) • Minutes of contact per ad-hoc session (Duration range: 10–120, with an average of 42 min; data current as of 11/18/19) • Dates of ad-hoc sessions (can occur any time during the participant’s 12 months in the study, i.e., in between intervention sessions as needed as well as after the six intervention sessions have been completed)
Scripting	Level of detail guiding interaction between the interventionist and the participant	• Semi-structured intervention with protocol provided • Some specific language provided with elaboration encouraged • Topics specified, but not necessary to discuss. Decision of which topics and when discussed is personalized based on the primary caregiver’s needs and preference • General guidelines provided
Sensitivity to participant characteristics	Extent to which participant background, experienceand abilities are incorporated in the delivery of intervention	• Outside of personalization of session content/topics, language preferences, literacy, visual supplements/augmented communication have not been incorporated (nor requested by participants)
Interventionist characteristics	Qualifications and training,concordance with participant characteristics	• Master’s degree or higher in marriage and family therapy, social work, counseling, or psychology • Personal or professional experience working with individuals with dementia and their families • Understanding and experience working with family systems • Training including: thorough understanding of treatment manual; shadowing of intervention sessions; and holding regular meetings to discuss intervention, available resources, and address questions as they arise for participants • Competence in tele-health delivery • Interventionist knowledgeable of cultural views and values of participants
Adaptability	Extent to which intervention can be modified. • What can be modified • On what basis modifications are made • When in the course of the study modifications can be made	• **What:** • Ad-hoc sessions may be added at any time • Duration of sessions • Location • Mode of delivery • Content • **On what basis:** • Participant request • Participant availability • Participant preference • Clinical judgment • **When:** • Any time while enrolled in study
Treatment implementation	**Treatment Delivery:**Documentation of interventionist compliance to intended treatment and modifications**Treatment Receipt:**Extent to which processes are implemented by participant and/or goals are met**Treatment Enactment:** Extent to which knowledge and skills acquired during treatment are applied inreal world settings outside of treatment	• Number and duration of sessions • Content delivered • Participant completion of review checklist at 4, 8, and 12 months • Participant self-report during sessions • Counselor appraisal of participant knowledge, skills, motivation, self-efficacy, and social support/integration via counseling notes • Semi-structured interviews conducted with purposively sampled primary caregivers at conclusion of participation
*Content and Goals*		
Treatment content strategies	Specific strategies aimed at improving outcomes	• Provision of information • Didactic instruction • Skill-Building techniques • Problem-Solving techniques • Stress management techniques • Facilitation of family and social support • Support provision for guilt and grief • Effective communication and conflict resolution skills
Mechanisms of action	Key processes, goals, or mediators of desired treatment outcomes	• Increased knowledge of dementia • Enhanced communication and conflict resolution skills • Acquisition of problem-solving skills • Increased prioritization and engagement in self-care • Increased caregiving self-efficacy • Increased caregiving competence • Increased comfort with residential long-term care • Increased social and family support

The RCTM is a semi-structured intervention and tailored to address the individual needs and concerns of a primary family caregiver. Over a four-month period caregivers engage in six consultation sessions conducted by a trained TC. Other family members may participate in the counseling sessions at the discretion of the primary caregiver. The TCs hold the first three RCTM counseling sessions weekly and the final three sessions monthly. Based on the caregiver’s preference, the RCTM sessions occur over the telephone or via secure web-based video conferencing. The sessions focus on the experiences of the caregiver, the care recipient, and potentially other family members following RLTC admission. Among the objectives for each session are the caregiver’s acquisition of information and strategies designed to deal with unique issues, such as distance caregiving. Specifically, the sessions are designed to:

- Establish a therapeutic rapport with the caregiver and the family;
- Provide a safe environment to explore stressors;
- Examine family relational dynamics as they relate to the RLTC placement decision itself, as well as the roles different family members play in the life of the caregiver and relative in RLTC;
- Identify new modes of communication to facilitate more effective interactions with other family members and care staff; and
- Identify effective ways to advocate for improved quality of care for and quality of life of their relatives in RLTC.

Throughout the RCTM counseling process/relationship, caregivers receive constructive feedback to help achieve their goals stated at the outset of the RCTM intervention. Session length typically ranges from 45 to 150 min, with an average session lasting 75 min.

The counseling sessions address several core questions, including:

- How does RLTC placement affect the caregiver or other family members participating in the counseling session?;
- What are the constraints and reasons for the way dementia care is provided in RLTCs?;
- How can this care be optimized?; and
- How can the family caregiver’s voice be heard when expressing the long-term care goals of their relative?

Critical stressors identified during the intake interview, crisis situations, and adaptation issues are incorporated to individualize participant counseling sessions, which use conversation, psychoeducation, and online information to support each caregiver.

All counseling components and content are designed to positively influence key outcome domains of RLTC admission for family caregivers: primary subjective stress; secondary role strain; care-related distress; and depressive symptoms (see below):

#### Psychoeducation

Education on how dementia affects the brain and behavior, personality, and cognition is provided to explain the changes the relative is currently experiencing and may experience in the future. There is a focus on the biological basis for why these changes occur, emphasizing that they are not under the relative’s control.

#### Promotion of communication

The objective of this component is to strengthen the caregiver’s skills in understanding other family members’ perspectives and to establish positive and collaborative relationships with RLTC staff. In addition, TCs introduce strategies for conflict resolution to empower caregivers and address issues before they become significant conflicts. Transition counselors offer ideas for specific activities designed to engage their relative during visits as well as support the caregiver’s ideas and creativity to meet the changing needs of their relative.

#### Problem-solving

Individual and family counseling sessions help caregivers divide potentially overwhelming problems into manageable components and direct the caregiver or other family members to (in) formal services available within the facility and in the community (e.g., ombudsman).

#### Care recipient behavior management strategies

Instruction focuses on understanding the potential causes of dementia-related behavior (e.g., aggression, repetitive questions, or taking other residents’ property) and determining ways to address the cause when possible. Instruction focuses on the acquisition of skills and strategies to manage caregiver reactions to unpredictable behavior (using elements of evidence-based interventions such as the Savvy Caregiver Program and strategies provided by the Alzheimer’s Association) [63, 64].

#### Concrete planning

This component explores goals to optimize personal and socioemotional care assistance for relatives in RLTC. Transition counselors collaborate with family caregivers to develop strategies to secure support from other family members and facility staff.

#### Making families aware

Caregivers acquire knowledge about the rehabilitative treatments used in RLTCs to effectively manage dementia symptoms (e.g., depression, agitation, etc.) and to determine whether such treatment approaches are available and delivered in the relatives’ RLTC setting. Caregivers also learn about the differences in levels of care and types of support found in different care settings (e.g., assisted living versus memory care).

#### Emotional health and stress management

Guilt, grief, and sources of stress are explored to help understand and improve emotional well-being. Validation, normalization, and reframing are employed to help reduce guilt, grief, and stress. The introduction of stress management techniques and relaxation exercises also aims to ameliorate caregiver stress. Family members further learn how their caregiving roles change when their relative moves to residential care.

Ad hoc sessions provide ongoing, informal counseling outside of the six scheduled sessions and are offered on the telephone, via secure web conferencing, or via email with a TC at the request of the family caregiver. This makes it possible for the TC to respond to the effects of the changing nature of ADRD; changes in the RLTC environment, services, and policies; and crises as they occur.

The TCs each have professional and personal experience with individuals with memory loss and their families. The required qualifications for the TC role are a Master’s degree or higher in marriage and family therapy, social work, counseling, or psychology as well as professional or personal experience working with individuals with dementia and their families. Specific characteristics of the TCs that are useful include knowledge of family systems and dynamics; understanding of dementia; ability to build personal rapport with participants; problem-solving skills; and extensive knowledge of communication styles and conflict resolution.

One TC holds a master’s degree, is licensed as a marriage and family therapist, and has extensive clinical experience working with older adults and their families. Another TC holds a PhD in clinical psychology and has years of experience working with adults and families in research interventions. Prior to the RCTM, the clinical expert who helped develop the intervention had expertise in working with family systems and found it important to have this understanding as a foundation to deliver the RCTM. One TC was hired at the outset of the study and the previous clinical expert was available to coach her on the intervention components and delivery as needed and to review the treatment manual used to guide the delivery of the RCTM intervention. The second TC was hired 1 year into the study due to the level of interest and enrollment on the part of caregivers. The first TC trained and mentored the second TC through each session initially in order to standardize implementation, information, and resources offered. Training included having the first TC reviewing the treatment manual with the second RC, having the 2nd TC shadow the first TC’s sessions, and both meeting regularly to discuss specific questions, available resources, and the intervention in detail. Training of the TCs lasted approximately 2 months each.

This intervention is delivered in a “tele-health” format: either via telephone or secure web-based conferencing. To date, the majority of participants (92%) choose to hold telephone-based sessions. For these reasons TCs should be competent in this mode of psychosocial/psychoeducational intervention delivery.

Following completion of the baseline survey, blinded graduate research assistants administer surveys to ADRD caregivers every 4 months thereafter for up to 12 months. Surveys continue to be administered to the caregiver whether the person with ADRD moves to another setting, passes away, or the participant is no longer the primary caregiver. A modified survey is sent to caregivers following the bereavement of the person with ADRD. At the time of each survey, data on placement transition and caregiver status is collected. Thus, up to four waves of empirical data (baseline/prior to intervention, 4-, 8-, 12-month intervals) will be available for participants who are not lost to follow-up.

To guarantee accuracy of treatment delivery, the PI and the previous clinical expert developed a detailed RCTM treatment manual based on preliminary evaluation of the intervention [62]. The treatment manual helps to ensure consistent implementation of the RCTM. The manual is an ongoing reference that provides a stepwise timeframe of delivery activities. The manual has also served as a training tool for the TCs, helping to enhance the consistency of the RCTM clinical approach and strategy.

Throughout delivery of the RCTM, the TCs maintain a detailed contact log and counselor notes to document the frequency, duration, and clinical content of each RCTM session; this serves as a means to assess treatment receipt [65] and allows the research team to track administration of the RCTM. Contact logs also help the team to document any deviations from the multi-session protocol of the RCTM. Also, as recommended by investigators of other evidence-based, ADRD caregiver interventions (e.g., REACH II) [66], feedback from caregivers themselves in the RCTM treatment condition helps the investigators further ascertain treatment receipt. Specifically, caregivers’ perceptions of the RCTM are assessed using the RCTM review checklist: a close-ended 22-item survey that asks caregivers to rate their experiences with various facets of the intervention using Likert scale items (1 = strongly disagree to 5 = strongly agree). This checklist is administered at each follow-up (4-, 8-, and 12-months) to caregivers who are randomly assigned to receive the RCTM intervention by a RC.

In order to elicit feedback from caregivers’ on their overall experiences with the RCTM intervention, an open-ended question is included on the RCTM review checklist. The open-ended responses yield qualitative data for why family caregivers felt individual or family counseling was helpful and any other comments they had about the RCTM, including aspects of the intervention they found most effective and suggestions for improvement and topics that may be important to include in future sessions.

Up to 30 semi-structured interviews with ADRD caregivers in the RCTM treatment condition will also take place. A graduate research assistant conducts these interviews within a 3-month period following completion of the participant’s final 12-month follow-up survey of the RCTM. The PI and TCs identify candidate caregivers throughout the course of the study who completed the RCTM and their 4-, 8-, and 12-month RCTM review checklists. Our initial plan was to select 15 ADRD caregivers who had an average review checklist score between 4 and 5 (agree and strongly agree, suggesting high perceptions of intervention utility) over the 12-month data collection period and 15 ADRD caregivers who endorsed total average review checklist scores below 3 (neutral or lower). However, the vast majority of participants provide review checklist scores of 4 and above in their appraisal of the RCTM intervention. For this reason, we widened our selection criteria for the latter category to include those who indicated a review checklist average score of below 4. To date, a total of five participants fall into the below 4 category. Although we may not obtain 15 interviews in the below 4 category, we will continue to pursue a total of 30 post-evaluation interviews. In addition to selecting higher and lower average review checklist scores, a stratified purposive sampling approach is applied: the PI and TCs purposively identify caregivers of varying kin relationship (spouse vs. adult child) and length of stay in the residential care setting.

The open-ended responses of the semi-structured interviews provide in-depth information on why and how dementia caregivers felt the RCTM counseling sessions influenced ADRD caregivers’ strain and adaptation to the residential care transition. A graduate research assistant conducts and digitally records the post-RCTM interviews over the telephone. A professional service transcribes interview recordings into a Microsoft Word file for subsequent analysis in NVivo 12. To date, the graduate research assistant has conducted 27 post-RCTM semi-structured interviews (see Supplementary File for interview guide).

We have adopted several strategies to address attrition bias. If ADRD caregivers wish to withdraw from the RCTM but agree to complete follow-up surveys, we continue routine follow-up data collection. Several steps also enhance study retention. A RC and graduate research assistants administer baseline and follow-up surveys in a format that is convenient to ADRD caregivers (via online or mail survey; a telephone survey option is also available). Baseline surveys usually take no more than 60 min; follow-up surveys typically last 45 min. Study staff provide weekly follow up reminders to enhance survey completion. Following completion of the 4-, 8-, and 12-month surveys, the TCs call participants to ask “how things are going” and offer the opportunity for control participants to feel connected to the overall study by offering information and referrals. A bi-annual project newsletter is sent to all participants to provide study updates and maintain rapport. Participants receive $25 following the completion of each baseline, 4-, 8-, and 12-month and final qualitative interview to compensate for their time and effort. Following completion of the final 12-month assessment, participants receive a call from one of the TCs and a handwritten note from the study team to thank them for their study participation.

The measures selected for the RCTM evaluation have strong psychometric properties, sensitivity to change, and are relevant to the RLTC transition as established in the SPM-RC [16, 67]. General measures of ADRD caregiver stress and negative mental health are also included. Caregivers complete measures at each time point (with the exception of context of care items, which are collected at baseline only).

#### Context of care

Geographic location is collected upon study enrollment. Baseline context of care variables include caregiver and care recipient demographics. Additionally, information is collected regarding the duration and extent of the care recipient’s memory problems, type of dementia diagnosis, Medicaid status, living arrangement/type of RLTC setting, and time since RLTC admission. Living arrangement of the care recipient is included at each time point; if a move from the current RLTC setting occurs, the date of the move and type of RLTC setting moved into is recorded. An overall rating of the RLTC setting’s care as perceived by the caregiver is assessed at each time point [68].

#### Primary objective stressors

Primary objective stressors include the care recipient’s dependence on assistance with six *activity of daily living (ADL) tasks* and six *instrumental activity of daily living (IADL) tasks* [69–71]. An 8-item scale assesses the intensity of the care recipient’s memory loss, communication deficits, and recognition failures at each time point (*memory impairment*) [46, 47]. Frequency of care recipient (CR) *behavior problems* is measured with the Revised Memory and Behavior Problems Checklist (R*-*MBPC) [72], which lists 24 common problems manifested by dementia patients.

#### Resources

*Socioemotional support* is measured on a five-item scale to assess the affective assistance provided to the caregiver by relatives or friends at each time point [46, 47]. Four subjective questions are included to assess caregiver health based on the Resources for Enhancing Caregiver Health II protocols [73]. Primary caregivers are asked to identify, from a fixed list of options, the number and frequency of community-based or health services used in the past 4 months [74]. Caregivers’ functional dependency is assessed by completing the ADL and IADL measures described above. We also administer the Self-Administered Comorbidity Questionnaire [75], which is a validated measure that collects information on the number and severity of comorbid conditions of caregivers. An 8-item measure of *caregiver self-efficacy* is included [76, 77]. Caregivers’ *sense of competence* is measured with the 7-item Short Sense of Competence Questionnaire (SSCQ), which assesses individuals’ sense of capability in providing assistance to a relative with ADRD [78–80]. The perceived *closeness of the relationship* with the care recipient in RLTC is also measured [16].

#### Primary subjective stress during residential care placement

A 7-item measure of *care-related strain* that assesses the stress family caregivers perceive as a result of having a relative in residential care is included as is a single-item measure of the caregiver’s difficulty in dealing with the relative’s mental state [16]. A 7-item version of the Zarit Burden Interview that captures relevant sources of emotional distress during a relative’s placement in RLTC is also included [18, 81–83].

#### Secondary role stressors

Secondary role stressors measure the adjustment of both the resident and caregiver to the new RLTC setting and/or shifting responsibilities in the caregiver-care recipient relationship that occur due to RLTC. Caregivers rate both their *relative’s level of adjustment* to the residential care setting, as well as their *own adjustment* on a pair of single items [16].

#### Residential care stress

Several measures examine caregivers’ perceptions of stress related to residential care placement [16, 40, 84]. *Family caregivers’ perceptions of staff communication with resident’s families* indicate how well family caregivers perceive the treatment their relatives receive when visiting the RLTC setting. *Staff support for families* ascertains family caregivers’ appraisals of the degree of socioemotional support they receive from RLTC staff. Caregivers are also asked to report the number of *positive and negative interactions* they have with their relative, RLTC staff, and other family members. The *Family Involvement Interview* (FII) is used to assess the range and frequency of family involvement in RLTC settings (e.g., ADLs, IADLs, socioemotional support, monitoring and discussing care with staff, directing care). *Visiting patterns* (e.g., length of time visiting) and *frequency of visits* (e.g., daily, weekly) are also collected.

#### Caregiver depressive symptoms

Caregivers’ *negative mental health* is measured with the Center for Epidemiological Studies-Depression scale [85] and the Mood Assessment Scale [86, 87].

### Analysis

Following the completion of surveys, the graduate research assistants download the survey data to a secure server. The graduate research assistants clean the data and create a master data file preserving a copy of the raw data. To conduct analyses, graduate research assistants will create an analytic dataset from the master data files. Analytic code, output and data will be saved for all analyses. The project biostatistician and the PI will supervise all empirical analyses. The PI in collaboration with the graduate research assistants will conduct thematic analysis of qualitative data collected in the treatment fidelity and post-RCT embedded procedures.

Intensive longitudinal analysis procedures (multilevel regression analyses of outcome and growth curve modeling) will be utilized to capitalize on the randomized controlled design and the multiple waves of data collected. The number of caregivers to be enrolled to address study hypotheses was determined using power analysis procedures that take into account the hierarchical analytic design of the study [88]. In this framework, the researcher identifies the Type I error rate (e.g., *p* < .05) to differentiate between a null and alternative test hypothesis, a suitable level of statistical power (.80 is considered an excellent power value), and the expected difference between the two study groups in order to determine the number of ADRD caregivers to enroll into the project. Relying on power estimations of behavioral interventions that are compared to usual care control groups [89], a “medium effect” size was determined in order to estimate a sample size appropriate to detect a group difference of 0.50 standard deviation units [62]. We also used a Bonferroni adjusted Type I error rate of .0125 (.05/4) to accommodate up to four primary outcome variables (primary subjective stress; secondary role strain; residential care stress; caregiver depressive symptoms), and we allowed for a conservative 10% loss to follow-up. With these specifications, an enrolled sample size of 240 ADRD caregivers (120 in each group) was sufficient to provide adequate statistical power. After attrition, this sample size will yield .87 power to detect a 0.50 effect size and .80 power to detect a slightly smaller effect size of 0.46 standard deviation units for the primary outcome: primary subjective stress during RLTC (i.e., the 7-item Zarit Burden Interview). This effect size could apply to covariate-adjusted mean differences at a particular follow-up or two linear slope differences of change across time between the intervention and control conditions.

As noted in various recommendations for mixed methods sampling, 30–40 participants is considered an adequate sample size for the semi-structured interview approach described here [90, 91]. Since sample size in qualitative research is based more on the richness and depth of open-ended data collected, it is possible to achieve the goals of the post-randomized controlled evaluation embedded component with a smaller number of semi-structured interviews. Given the expected number of ADRD caregivers in the RCTM treatment condition, we decided on up to 30 semi-structured interviews to ensure the richness of the qualitative data collected.

Data available at baseline, 4 months, 8 months, and 12 months will allow for individual growth curve models that examine change in ADRD caregiver outcomes [92, 93]. Multilevel analysis approaches are available that support growth curve modeling. In this context, growth curve modeling is an example of a two stage modeling process consisting of: 1) a within-subjects model across time, and 2) a between-subjects model that incorporates caregiver and care recipient covariates [94, 95]. The primary independent variable in the proposed investigation consists of an indicator variable for random assignment into the RCTM treatment condition or the attention care control. SAS (version 9.4) Proc Mixed [96] will be used to conduct these analyses, as it supports multilevel and growth curve modeling procedures.

The proposed analyses will provide in-depth tests of Specific Aims 1 to 3 (i.e., rates of change in primary subjective stress, secondary role strains, residential care stress, and caregiver depressive symptoms) over a 12-month period. In one set of outcome evaluations, the baseline value will be included as a covariate, and time will be centered at 4-months post-baseline. This scales the intercept effect to be a main effect of RCTM group assignment and allows the RCTM treatment and the attention control groups to have different 4-, 8-, and 12-month change trajectories, or a RCTM treatment*time interaction effect. After establishing that the individual growth parameter estimates have significant variance (*p* < .05) around the mean trajectories of change in key dependent variables, an RCTM treatment vs. control group indicator will be added as the key independent variable to predict intercepts and rates of change in outcomes. Additional analyses will determine if covariates (e.g., context of care indicators, primary objective stressors, and resources) significantly vary (*p* < .05) across the RCTM treatment and control groups at baseline and over time via growth curve modeling procedures. If statistically significant (*p* < .05) variations between the RCTM treatment and control groups are found, initial status and rate of change parameters for these covariates will be included in all tests to provide further statistical control.

In addition to analyses of secondary Aims 1a and 1b (which will mirror the Specific Aims 1–3 analyses described above), empirical treatment fidelity data on variations of the RCTM (e.g., frequency and duration of counseling sessions) will be included in growth curve models as a series of post-hoc Specific Aims 1, 2, and 3 analyses. These analyses will explore the effects of variations in RCTM utilization on the hypothesized outcomes. A series of mediational models will also be tested to examine some of the hypothesized pathways of the SPM-RC as described in the conceptualization of the proposed project (e.g., whether RCTM assignment mediates the empirical relationships between care-related stressors and more global psychological and emotional outcomes on the part of family caregivers). As detailed by Selig and Preacher and other methodologists, mediational models appropriate for longitudinal data will be utilized [97]. These analyses will explore the empirical mechanisms that explain RCTM’s efficacy or lack thereof.

The goal of Specific Aim 4 analyses will be to determine how and why the RCTM results in benefits for dementia caregivers (or not). Open-ended data from the RCTM review checklists as well as the semi-structured interviews completed after RCTM intervention delivery will yield in-depth information on aspects of the RCTM intervention deemed helpful by participants; how counseling recommendations were utilized when interacting with the care recipient living in RLTC, staff, or other family members; and why dementia caregivers felt the RCTM helped them to experience reduced distress and improved well-being (or failed to do so). The graduate research assistants will independently read text to identify elements, or codes, that are common in the qualitative transcripts. The graduate research assistants will then combine these codes into more general “categories” which are compared, contrasted, and refined to identify themes that run throughout the qualitative data. nVivo 12 software will be utilized to facilitate the qualitative data analysis [98]. A consensus process featuring the first author and the graduate research assistants will occur, where codes, categories, and themes are discussed and a general agreement is reached as to how these qualitative elements emerge and are linked in conceptual patterns. All decisions will be logged in an audit trail. Expert co-authors/co-investigators will also discuss, review, and offer expert insights regarding the emerging qualitative findings during monthly meetings. The various levels of qualitative analysis proposed are anticipated to generate a rich exploration of the mechanisms of RCTM benefit and will also offer quality checks on the qualitative data collected. The thematic results will also be reviewed by RCTM study participants so that are meanings and conclusions are appropriate to the experiences of dementia caregivers (i.e., “member-checking”) [99, 100].

Mixed methods analysis will also take place [51, 52]. The qualitative data collected from the various “embedded” procedures (i.e., RCTM review checklists, post-RCTM semi-structured interviews) will be cross-tabulated with the longitudinal empirical data from the quantitative evaluation of the RCTM. Cross-tabulations of themes and categories with empirical data will allow for an examination of whether qualitative and quantitative findings converge or diverge. In the latter case, such trends can highlight additional queries to guide post-hoc analyses of the evaluation data to ascertain whether the RCTM lacks efficacy for certain outcome variables or there are potential reasons for the apparent lack of quantitative findings [51, 101, 102].

A data safety and monitoring plan (DSMP) is in place to ensure appropriate oversight of the research protocol and adverse event reporting procedures, if necessary. The Data Monitoring Officer (DMO) reviews data monitoring and safety activities annually during the 5-year project period. The responsibilities of the PI (who also has oversight for the data management and with the project biostatistician all data analyses) and a RC include the production of an administrative report that highlights study accrual. In addition, the PI and a RC provide information on any deviations from the approved protocol (e.g., adherence to study eligibility criteria) and any other issues related to the progress of the study. The DMO reviews the administrative report to ensure ongoing quality control. Following this review and the resolution of any concerns, the administrative report is sent to the Program Officer of the grant that supports this research (R01 AG04893) at the National Institute on Aging (NIA). In instances of unanticipated/adverse events, the UMN IRB is notified as soon as possible per the IRB’s standard policy and procedures.

Main outcome papers that address the study aims will not be disseminated until all data collection procedures are completed. However, analyses of treatment receipt, perceived utility, important aspects of intervention delivery, and other descriptive analyses have and will be disseminated prior to the main outcome papers. The project team aims to minimize the length of time between final data collection procedures and dissemination of final outcome papers to appropriate peer-reviewed journals. We anticipate a timeframe of 6 months between final data collection and peer-reviewed article submissions of RCTM outcomes. When manuscripts are published, the findings will be distributed to all research participants.

## Discussion

People live with ADRD from 4 to 8 years on average and up to 20 years following a diagnosis [103–111]. As the disease progresses, changes often occur in the course, provider, or setting of care (i.e., *care transitions*) [112–118]. Challenges during care transitions—ranging from lack of information to poor care coordination—contribute to negative health and service utilization outcomes for persons living with dementia, their family caregivers, and professional care providers if not prevented or navigated effectively [47, 116, 119–122]. Therefore, helping individuals, families, and organizations avoid or better navigate transitions may prove a more valuable strategy than other, more general approaches when improving long-term clinical benefits.

Important ADRD care transitions include dementia caregiving onset and the initial diagnosis of dementia; exacerbation of behaviors and community-based service use; hospitalization/hospital to home; and residential long-term care admission (the focus of the RCTM) [47, 121, 123–125]. The RCTM is different from many evidence-based caregiver intervention models because it targets a key transition in the course of the dementia caregiving trajectory, and as a result is a more focused and compact intervention model than other multi-component dementia caregiver interventions. Given the focus of the RCTM, it is anticipated that the RCTM will be among the first clinical interventions that adopt a family-centered focus to facilitate successful residential transitions for older persons with cognitive impairment.

The RCTM has undergone multiple phases of testing to develop a protocol that is clinically and conceptually tailored to facilitate families’ management of the RLTC transition for cognitively impaired relatives. This process of testing incorporated the principles and methods of the Science of Behavior Change (SOBC) [126]. The three steps of SOBC are identify, measure, and influence. Progression through these three steps can help investigators better understand why an intervention does or does not work. The PI’s early descriptive research on institutionalization and dementia caregiver adaptation identified that some family members experience negative emotional, social, and psychological effects of admitting a cognitively impaired relative to residential long-term care. These outcomes were often due to feelings of relationship impairment with persons with ADRD as well as challenges navigating and interacting with the long-term care environment itself [6, 18, 19, 127–130]. The 1st author and his colleagues used qualitative data syntheses as well as identification of existing tools [6, 13, 16, 42] to measure the emotional, social, and logistical threats to a relative’s nursing home adaptation as well as inform the content and structure of a potential intervention for dementia family caregivers experiencing this transition [130]. He and his current team intend to influence family caregivers’ emotional and psychological adaptation to a relative’s admission to RLTC through the RCTM, which has demonstrated initial promise via a pilot evaluation [62].

There are several important limitations to this protocol. Data collection is survey- and interview-based and more objective measures of stress were not considered. Still, self-report measures of subjective stress, depressive symptoms, and similar domains remain valid and cost-effective in the evaluation of dementia care interventions. The RCTM is targeted and delivered to the family caregiver; we do not incorporate other key stakeholders (e.g., residential care staff) in our intervention nor in our outcome measures. In future iterations of the RCTM we are planning to incorporate residential care staff into the intervention.

Elements of the RCTM make this intervention amenable to dissemination and implementation. The RCTM is not limited by geographic distance, allowing for flexible delivery. The individualized, tailored content of the RCTM that addresses key content areas and specific areas of need (regardless of time since admission to RLTC) is another feature that likely enhances its implementation potential as the intervention can meet the heterogeneous needs of families following RLTC admission of a relative. However, there are dissemination and implementation challenges. The current RCTM TCs are highly trained clinicians who hold advanced professional degrees/licenses. Whether facility staff or other professionals with less professional and clinical expertise can effectively deliver the RCTM is unknown and a major consideration as our implementation plan is refined and deployed.

## Data Availability

The de-identified data supporting our findings will be shared on the National Archive of Computerized Data on Aging (NACDA).

## Abbreviations

ADL: Activity of daily living
ADRD: Alzheimer’s disease or a related dementia
DMO: Data Monitoring Officer
DSMP: Data and safety monitoring plan
FII: Family Involvement Interview
IADL: Instrumental activity of daily living
IRB: Institutional review board
NH: Nursing home
NIA: National Institute on Aging
RCT: Randomized controlled trial
RC: Research coordinator
RCTM: Residential Care Transition Module
R-MBPC: Revised Memory and Behavior Problems Checklist
RLTC: Residential long-term care
SOBC: Science of Behavior Change
SPM: Stress Process Model
SPM-RC: Stress Process Model for Residential Care
TC: Transition counselor
UMN: University of Minnesota
